# Replication kinetics of pathogenic Eurasian orthohantaviruses in human mesangial cells

**DOI:** 10.1186/s12985-024-02517-5

**Published:** 2024-10-01

**Authors:** Lukas Boegelein, Pamela Schreiber, Alexandra Philipp, Christian Nusshag, Sandra Essbauer, Martin Zeier, Ellen Krautkrämer

**Affiliations:** 1 Department of Nephrology, University of Heidelberg, Im Neuenheimer Feld 162, D-69120 Heidelberg, Germany; 2 Department Virology and Intracellular Agents, Bundeswehr Institute of Microbiology, German Centre for Infection Research, Munich Partner Site, D-80937 Munich, Germany

**Keywords:** Orthohantavirus, HFRS, Acute kidney injury, Mesangial cells, Cell line

## Abstract

**Background:**

Eurasian pathogenic orthohantaviruses cause hemorrhagic fever with renal syndrome (HFRS) characterized by acute kidney injury (AKI). The virulence of orthohantaviruses varies enormously and direct infection of different renal cell types contribute to pathogenesis. Glomerular mesangial cells play an essential role in the interplay between kidney cells and proper kidney function. Therefore, we analyzed the replication competence of different orthohantavirus species in primary mesangial cells and a mesangial cell line.

**Methods:**

We tested the suitability of the mesangial cell line CIHGM-1 (conditionally immortalized human glomerular mesangial cells) as cell culture model for orthohantavirus kidney infection by comparison with primary human renal mesangial cells (HRMCs). We analyzed infection with high pathogenic Hantaan virus (HTNV), moderate pathogenic Puumala virus (PUUV) and non-/low-pathogenic Tula virus (TULV).

**Results:**

Effective viral spread was observed for PUUV only, whereas infection with HTNV and TULV was abortive. However, in contrast to TULV, HTNV exhibits an initially high infection rate and declines afterwards. This replication pattern was observed in HRMCs and CIHGM-1 cells. Viability or adhesion was neither impaired for PUUV-infected CIHGM-1 nor HRMCs. A loss of migration capacity was observed in PUUV-infected CIHGM-1 cells, but not in HRMCs.

**Conclusions:**

The identification of differences in the replication competence of pathogenic orthohantavirus strains in renal mesangial cells is of special interest and may provide useful insights in the virus-specific mechanisms of orthohantavirus induced AKI. The use of CIHGM-1 cells will facilitate the research in a relevant cell culture system.

## Introduction

Involvement of the kidney is a hallmark of several infectious diseases [1–4]. The underlying mechanisms are not completely understood. Immune-mediated as well as direct effects by infection of kidney cells may contribute to the clinical picture [5–8]. Complex interplay between different cell types in the kidney is required for proper function [9] and targeting of renal cells by viruses may interfere with these processes.

Hemorrhagic fever with renal syndrome (HFRS) caused by infections with Eurasian orthohantaviruses is characterized by its specific renal manifestation [3, 10, 11]. Despite high genetic similarity of orthohantaviruses, the symptoms and disease severity vary enormously between species and members. In contrast to Eurasian orthohantaviruses, species in the Americas cause hantaviral cardiopulmonary syndrome (HCPS) [12]. The pathogenicity factors for the pronounced organ-specific manifestation in HFRS and HCPS are not identified so far. Glomerular and tubular cells are affected in HFRS as demonstrated by light and electron microscopy analysis of kidney specimens [13–18]. Kidney injury is characterized by endothelial and epithelial morphological changes, thickening of the glomerular basal membrane, foot process effacement of podocytes as well as hypercellularity and expansion of the mesangium. In addition, release of soluble factors by kidney and immune cells influences kidney function, repair mechanisms, and disease severity [6–8, 19–27]. Especially, mesangial cells may modulate the glomerular response to infection by changing the microenvironment and crosstalk between cell types [28]. The role of mesangial cells in glomerular function is strikingly demonstrated in IgA nephropathy (IgAN), which is a common cause of glomerulonephritis and renal failure. Studies from patients suffering from IgAN revealed that mesangial cells secrete soluble factors, which mediate reduced adhesive and migratory capacity of podocytes leading to podocyte dysfunction and loss [29, 30]. Identification of target cells and soluble mediators contributing to infection-induced kidney injury is an important step in orthohantaviral research. Viral antigens and genomes of Eurasian orthohantaviruses are detected in renal tissue and in vitro infection studies identified different renal cell types as target cells for pathogenic orthohantaviruses [31–35]. Kidney samples of HTNV or PUUV infected patients and samples from PUUV infection model with macaques revealed infection of tubular epithelial cells and the presence of infected cells in the glomerular apparatus without specifying the glomerular cell type [31–37]. The identification of target cells within the glomeruli is demanding. HFRS kidney samples are rare and sampled from fatal cases or late during the clinical course and may not reflect the situation early after infection. Therefore, cell culture experiments in vitro will be helpful to examine orthohantaviral pathogenesis. Despite the organ-specific manifestation of HFRS, in vitro studies using relevant human renal cell types are still sparse. The first in vitro study in renal cells by Temonen et al. identified podocytes and glomerular mesangial cells as permissive for infection with PUUV [35]. Additional in vitro studies demonstrate that pathogenic orthohantaviruses infect tubular epithelial cells, podocytes, and glomerular endothelial cells, but the infection of tubular epithelial and glomerular endothelial cells with the non-/low-pathogenic TULV is absent or abortive [34, 38, 39]. Infected kidney cells exhibit several morphological and functional consequences. Cytoskeletal rearrangement, disruption of cell-to-cell contacts, impaired adhesion and motility were observed in renal cells, which may contribute to acute kidney injury [34, 40]. These effects are cell-type specific [39, 41]. In vitro cell culture studies in relevant target cells provide useful insights in the pathogenesis of AKI in infectious diseases. However, organ-specific epithelial and endothelial cells are highly specialized and differentiated cell types. The cultivation in vitro is difficult, and immortalization often changes their characteristics [42, 43].

We identified primary human mesangial cells as target cells of pathogenic orthohantavirus PUUV, whereas infection with non-/low-pathogenic TULV is poor and abortive [44]. Despite presence of receptors for PUUV and TULV, integrin α_v_β_3_ and β_1_, respectively, the permissiveness differs between the two species. To perform further studies concerning replication kinetics and to identify restriction factors, a mesangial cell line would be favorable for orthohantavirus studies. We used the immortalized cell line CIHGM-1, which is described to be an adequate in vitro cell culture model for human mesangial cells. It possesses the typical protein expression profile and morphological and functional attributes that are characteristic for glomerular mesangial cells [45]. Therefore, we evaluate the suitability of the mesangial cell line CIHGM-1 as in vitro cell culture model for infection of human mesangial cells by orthohantaviruses with different virulence.

## Materials and methods

### Cells

Conditionally immortalized human glomerular mesangial cells (CIHGM-1) were maintained in RPMI1640 medium [45]. Conditionally immortalized human podocytes (CIHP) were kindly provided by Jochen Reiser [46] and were cultured in RPMI1640 medium supplemented with 10% FCS and 1% insulin-transferrin-selenium (Invitrogen, Thermo Fisher Scientific, Waltham, MA, USA). CIHGM-1 cells and CIHP were conditionally immortalized with the temperature-sensitive SV40 large T antigen, and differentiation of CIHGM-1 cells and CIHP was induced by shifting to 37 °C. Primary human renal mesangial cells (HRMC) were obtained from Sciencell (San Diego, CA, USA) and maintained in mesangial cell medium (Sciencell). Vero E6 cells from African Green Monkey kidney were maintained in DMEM supplemented with 10% FCS. Human umbilical vein endothelial cells (HUVEC) were purchased from Promocell (Heidelberg, Germany) and maintained in endothelial cell growth medium. Culturing of CIHGM-1 cells, CIHP and HRMCs was performed without adding antibiotics. Cells were routinely tested for mycoplasma contamination via PCR (Venor^®^GeM Classic, Minerva Biolabs, Berlin Germany).

### Viruses and infection

Puumala virus (PUUV) strain Vranica, Tula virus (TULV) strain Moravia, Hantaan virus (HTNV) strain 76–118 were propagated and titrated by single round infection assay via intracellular immunofluorescence for N protein on Vero E6 cells. Infection of different cell types (10,000 cells/cm^2^) was performed with equal volumes of viral stocks corresponding to an MOI of 1 for infection of Vero E6 cells allowing the comparison of infection rates between cell types. After one hour the viral inoculum was removed and fresh medium was added after a triple wash with medium. Work with infectious viruses was performed in biosafety level 2 and 3 containment facilities. Release of particles was determined by reinfection of Vero E6 cells in a single round infection assay [47]. In brief, titers of infectious units (IU) were measured by inoculation of 1.5 × 10^4^ Vero E6 cells with 10 µl cell-free supernatant from infected mesangial cells for one hour. Subsequently, Vero E6 cells were washed three times with medium and fresh medium was added. Infected Vero E6 cells were quantified, and titers were calculated as IU per ml supernatant. Infected cells were quantified by detection of N protein expression via immunofluorescence early after inoculation during the first round of infection (48 h post inoculation). Viral titer was calculated using the formula: Titer (IU/ml) = N_IC_ x 1000/V_SN_. N_IC_: number of infected cells; V_SN_: volume of supernatant in µl added to Vero E6 cells.

### Immunofluorescence and Western blot

For immunofluorescence, cells were grown on coverslips and fixed with 3% paraformaldehyde. The following primary and fluorescently-labeled secondary antibodies were used for staining: mouse anti-α-smooth muscle actin (α-SMA) (clone 1A4, Sigma, St. Louis, MO, USA), mouse anti-synaptopodin (clone D-9, Santa Cruz, Dallas, TX, USA), mouse anti-cytokeratin 18 (CK18) (clone RGE-53, Millipore, Burlington, MA, USA), rabbit anti-fibronectin (Sigma), mouse anti-CD31 (Dako Agilent, Santa Clara, CA, USA), mouse anti-N protein PUUV (A1C5, Progen, Heidelberg) for the detection of N protein of PUUV and TULV, mouse anti-N protein HTNV (B5D9, Progen). Cell nuclei were stained by Hoechst 33,342 (Invitrogen, Waltham, MA, USA). Images were taken using an Axiocam 506 mono camera attached to an Axio Observer.D1 inverted microscope (Carl Zeiss, Wetzlar, Germany). For Western blot analysis, equal volumes of cellular lysates were analyzed. The following primary antibodies were used: rabbit anti-HTNV N protein for the detection of HTNV N protein and rabbit anti-PUUV N for the detection of N protein of PUUV and TULV. Both antibodies were generated in-house with full length recombinant N proteins of HTNV and PUUV [48]. Analysis of tubulin with mouse anti-α-tubulin (Sigma) on the same membrane served as loading control. Near infrared fluorescent dye (IRDye)-conjugated secondary antibodies and the Odyssey CLx infrared imaging system (Li-Cor, Lincoln, NE, USA) were used for detection.

### Flow cytometry

Surface expression of orthohantaviral receptors was analyzed by flow cytometry. CIHGM-1 cells were washed with PBS, scraped, and stained with phycoerythrin (PE)-conjugated mouse anti-integrin α_V_β_3_ antibody (clone LM609, Millipore) or PE-conjugated mouse anti-integrin β_1_ antibody (clone P5D2, R&D Systems, Minneapolis, MN, USA) together with allophycocyanin (APC)-conjugated anti-CD55 (clone IA10, BD Pharmingen, NJ, USA). Controls were incubated with the corresponding isotype antibodies. Flow cytometry analysis was done after one hour of incubation on ice with FACSCalibur (BD Pharmingen). Debris and non-viable cells were identified and excluded from analysis by forward and side scatter and by using Via-Probe™ Cell Viability Solution (BD Pharmingen) according to manufacturer’s instructions.

### Motility assay

Uninfected and infected CIHGM-1 cells (10,000 cells/cm^2^) were seeded on µ-slide 8-wells (Ibidi, Gräfelfing, Germany). At six days post infection (dpi), cells were subjected to live cell imaging for six hours by JuLi Smart Fluorescence Cell Imager (Digital-Bio, NY, USA). In each experiment, the covered distances were monitored for 30 cells by the ImageJ manual tracking plugin (Ibidi). Statistical analysis of motility was done by using the chemotaxis tool plugin (Ibidi).

### Adhesion assay

At day six post infection, uninfected or infected CIHGM-1 cells were added in a 96-well microtiter plate (10,000 cells/well) and left to adhere for 30 min at 37 °C. After a triple washing with PBS, adhered cells were fixed, stained with Sapphire700 (Li-Cor) and DRAQ5 (BioStatus, Shepshed, United Kingdom) and quantified via scanning with Odyssey CLx infrared imaging system (Li-Cor).

### Viability

CIHGM-1 cells were infected with PUUV and lysed on 6 dpi. Viability of uninfected and infected cells was determined by measuring the amount of ATP using CellTiter-Glo^®^luminescent cell viability assay (Promega, Walldorf, Germany).

### Statistical analysis

Statistical testing was performed using Prism 5.0 (Graphpad Software Inc., San Diego, CA, USA). Normal distribution was tested by the Shapiro-Wilk test. Results of uninfected and infected cells were compared using two-tailed Student’s t-test. p values of < 0.05 were considered significant. ns: not significant; * *p* < 0.05. Data are given as mean and standard deviation (mean ± SD).

## Results

### Marker and receptor expression of CIHGM-1 cells

**Fig. 1:**
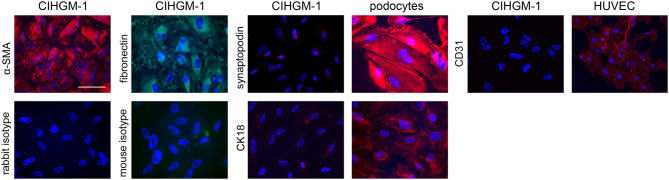
Expression of marker proteins in CIHGM-1 cells. Cells were stained for α-SMA, fibronectin, synaptopodin, CK18 and CD31. Podocytes served as positive control for the podocyte-specific protein synaptopodin and the epithelial marker CK18, HUVECs were stained as control for the expression of the endothelial marker CD31. Scale bar: 100 μm

Cells were analyzed for marker proteins by immunofluorescence (Fig. 1). They were positive for α-SMA and fibronectin, which are described as markers typically expressed by mesangial cells [45]. In contrast, no expression of the podocyte-specific protein synaptopodin, the endothelial marker protein CD31 or the epithelial marker CK18 was observed. The marker expression corresponds to the characteristic profile observed for mesangial cells.

**Fig. 2:**
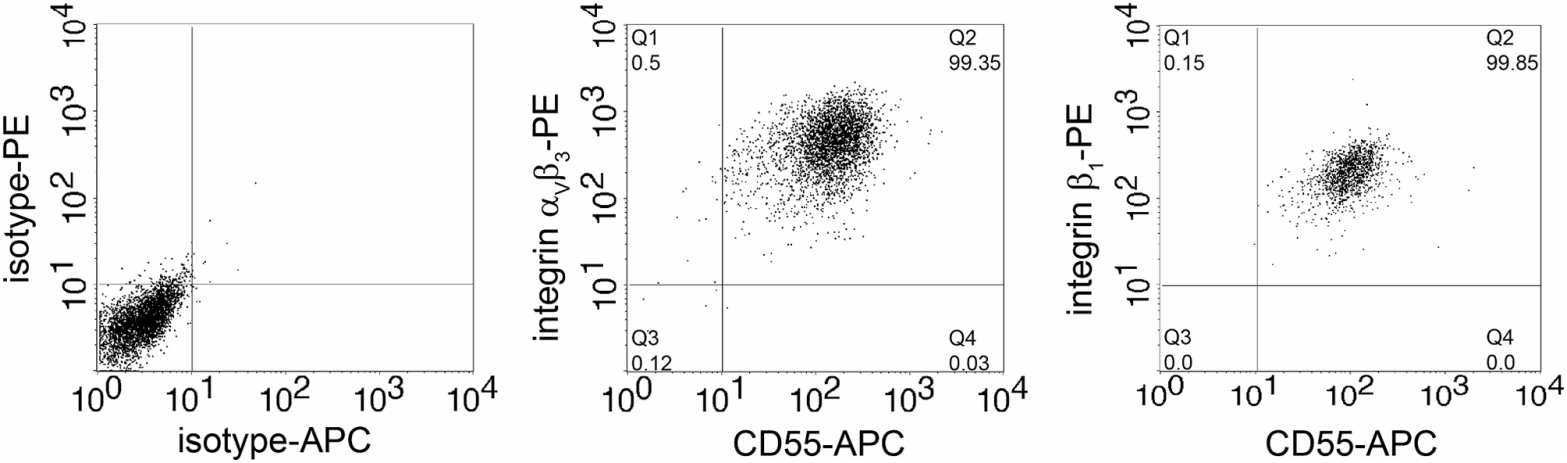
Flow cytometry analysis of surface expression of orthohantaviral entry receptors. CIHGM-1 cells were analyzed for the presence of integrin α_v_β_3_, integrin β_1_, and CD55 on the cell surface. Plots shown are gated on the viable cell population according to scatter profile and the exclusion of Via-Probe™ Cell Viability Solution. Quadrant statistics (Q1-Q4) indicate the percentage of cells in the respective quadrant

In the next step, we examined the surface expression of orthohantaviral receptors (Fig. 2). Integrin α_v_β_3_ and integrin β_1_ are described to mediate entry of pathogenic and non-pathogenic orthohantaviruses, respectively [49, 50]. In addition, CD55 (complement decay-accelerating factor, DAF) serves as co-receptor in orthohantaviral entry [51, 52]. More than 99% of cells are positive for surface expression of the respective integrin together with CD55. Our results demonstrate the expression of all three receptors on the surface of CIHGM-1 cells as observed for primary mesangial cells [44].

### Infection of CIHGM-1 cells with TULV and PUUV

**Fig. 3:**
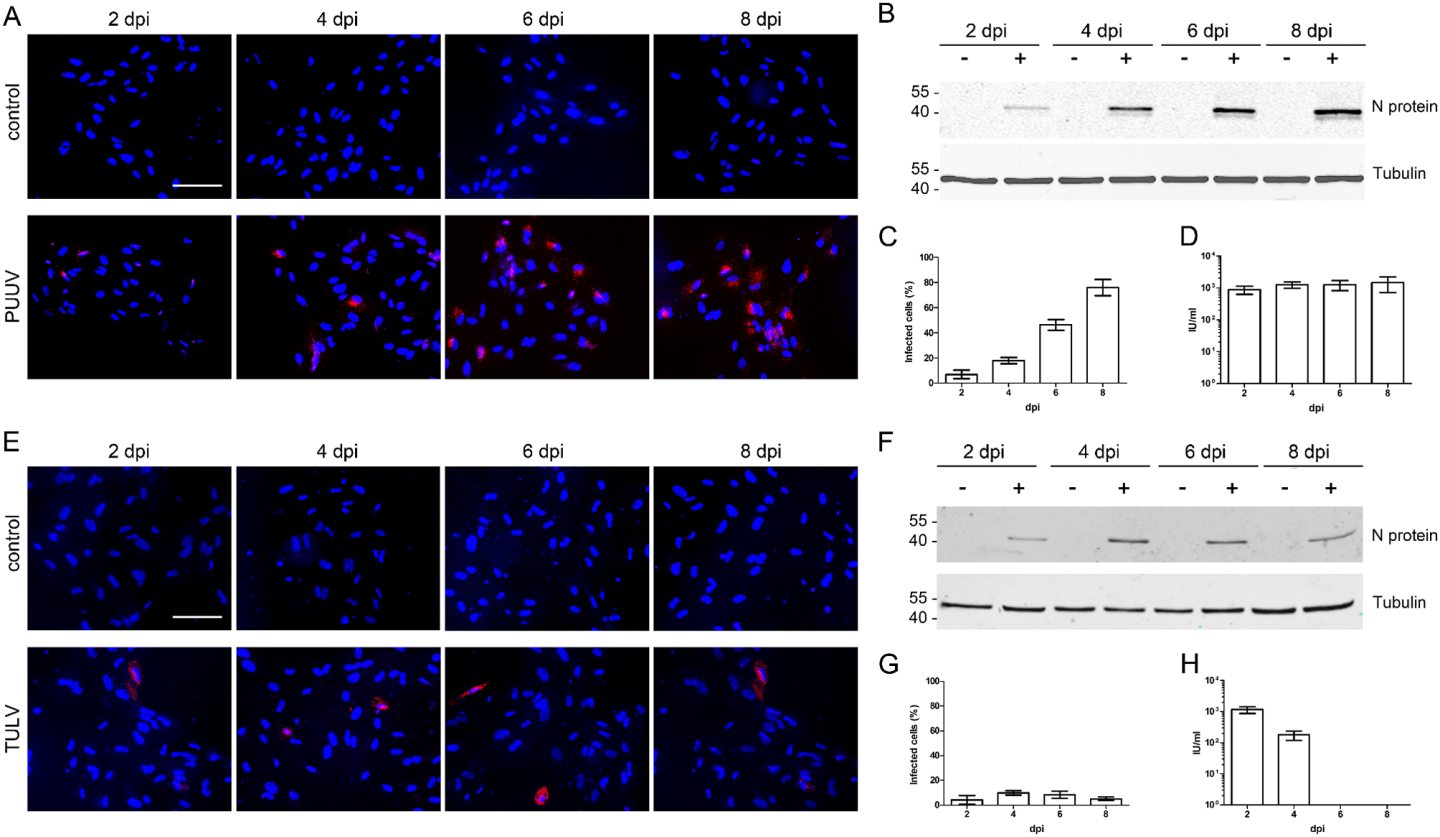
Infection of CIHGM-1 cells with PUUV (**A**-**D**) and TULV (**E**-**H**). Cells were inoculated at an MOI of 1. Infection was demonstrated by detection of N protein via immunofluorescence (scale bar: 100 μm) (**A** and **E**) and Western blot (**B** and **F**). Percentage of infected cells was quantified by counting N protein expressing cells (**C** and **G**). Infectious units (IU) in supernatants of CIHGM-1 cells were quantified by single-round infection assay on Vero E6 cells (**D** and **H**). Three independent experiments were performed. Mean ± SD are shown

Our studies in primary human renal mesangial cells revealed the productive and robust infection with PUUV but a low and abortive infection for TULV [44]. To evaluate the suitability of the mesangial cell line compared to primary cells, we incubated CIHGM-1 cells with PUUV and TULV (Fig. 3). As observed for HRMCs, PUUV replicated efficiently in CIHGM-1 cells as demonstrated by an increase of N protein expressing cells from 6.91% ± 0.33% (mean ± standard deviation) at day two post infection to 76.06% ± 6.59% at day eight and the presence of N protein in cellular lysates (Fig. 3A, B and C). In contrast, less TULV N protein was detected in lysates and only a small percentage of cells was positive for TULV N protein at 2 dpi and infection peaked at day 4 with 9.79% ± 1.71% infected cells (Fig. 3E, F and G). Release of infectious TULV particles was quantified by infection of Vero E6 cells with supernatants derived from mesangial cells and infectious units were observed at day two and four post infections, but not at later time points (Fig. 3H). In PUUV-infected cells, infectious virus was also released at day six and eight after inoculation as demonstrated by reinfection assay (Fig. 3D).

### Functional consequences of PUUV-infection in CIHGM-1 cells

**Fig. 4:**
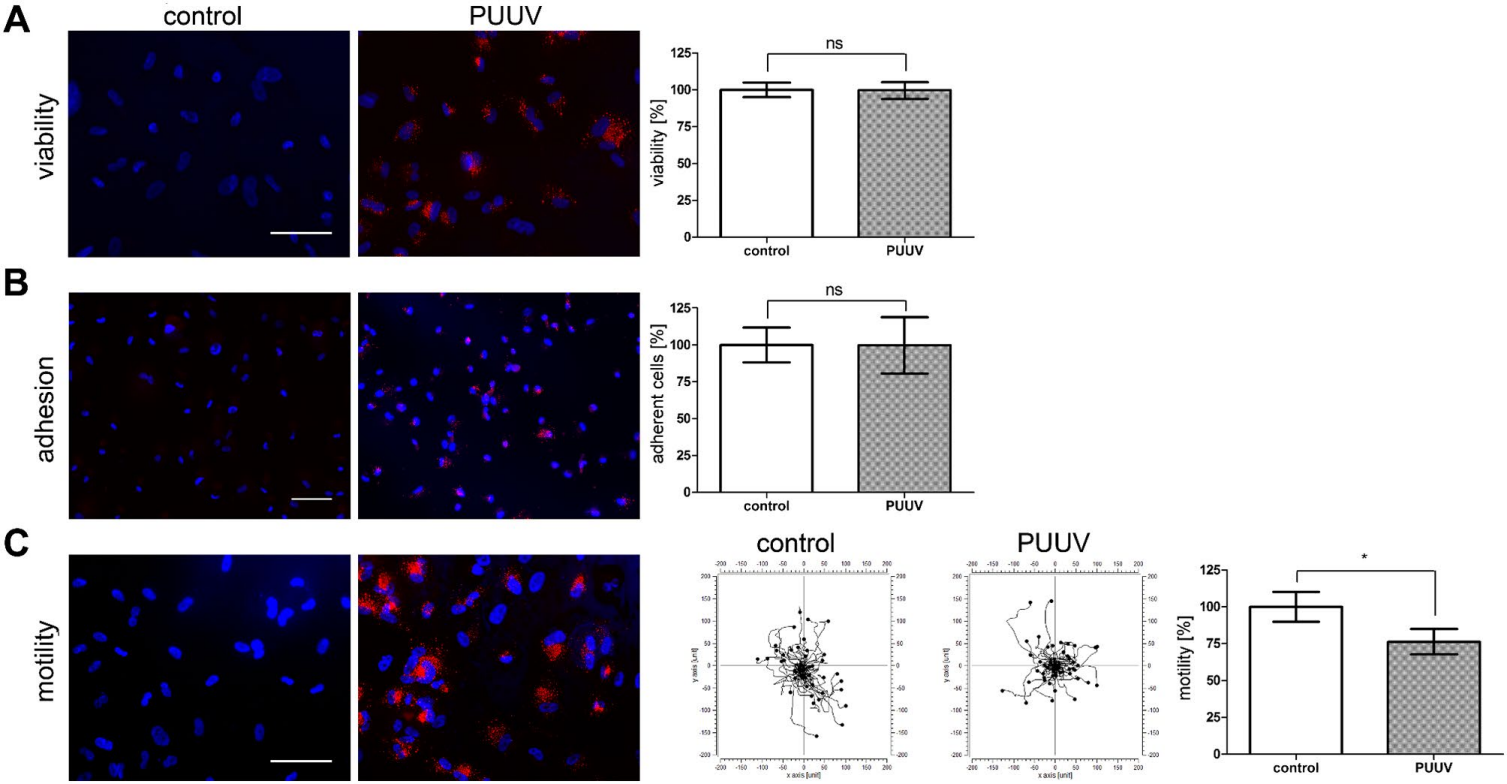
Functional analysis in PUUV-infected CIHGM-1 cells. Viability (**A**), adhesion (**B**), and motility (**C**) were analyzed in PUUV-infected CIHGM-1 cells at day six post infection (scale bar: 100 μm). Three independent experiments were performed. Uninfected control cells were set to 100%. Mean ± SD are shown

The orthohantaviral infection of renal cells does not affect viability of cells but leads to different cell-type specific functional consequences [40, 41]. Infected podocytes and tubular epithelial cells exhibit impaired motility and adhesion. These functional effects were not observed in primary human mesangial cells [44]. Therefore, we tested viability, motility, and adhesion capacity of CIHGM-1 cells infected with PUUV (Fig. 4). More than 98% of cells were infected as demonstrated by immunofluorescence staining for N protein in each experiment. Infection of CIHGM-1 cells does neither affect viability nor adhesion (Fig. 4A and B). However, infected CIHGM-1 cells show a mean reduction in motility of 23.69% ± 8.61% compared to uninfected cells (Fig. 4C).

Together, the results for permissiveness and replication of TULV and PUUV in CIHGM-1 cells correspond to the observations made in primary mesangial cells, whereas slight dissimilarities were observed in the functional effects.

### Infection of CIHGM-1 cells and HRMCs with HTNV

**Fig. 5:**
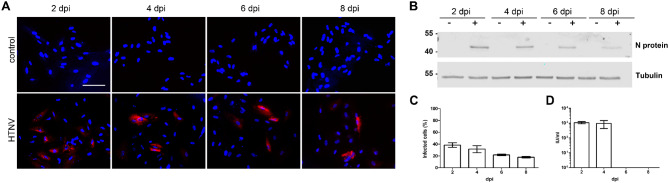
Infection of CIHGM-1 cells with HTNV. Viral inoculum was added at an MOI of 1. Infection was monitored via detection of N protein by immunofluorescence (scale bar: 100 μm) (**A**) and Western blot (**B**). Replication was analyzed by counting N protein expressing cells at the indicated time points (**C**). Release of infectious units (IU) was determined by single round infection assay by incubation of Vero E6 cells with cell-free supernatants derived from CIHGM-1 cells at the indicated time points (**D**). Three independent experiments were performed. Mean ± SD are shown

We observed strong differences in the permissiveness of mesangial cells between pathogenic PUUV and non-/low-pathogenic TULV. Therefore, we analyzed the infection of mesangial cells with the high pathogenic orthohantavirus HTNV (Fig. 5). At day two, we detected N protein in cellular lysates of CIHGM-1 cells by Western blot analysis and a mean initial infection of 38.37% ± 3.79% of cells as quantified by immunofluorescence (Fig. 5A, B and C). Afterwards, the number of infected cells declined with less than 20% of cells infected at 8 dpi. Despite a high percentage of initially infected cells, no spread of HTNV infection was observed in the mesangial cell line.

**Fig. 6:**
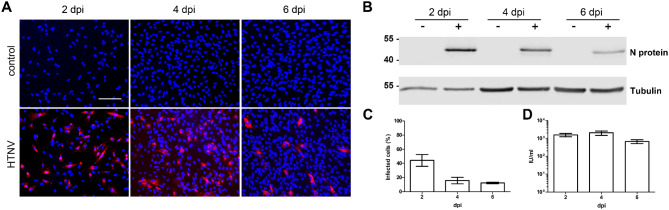
Infection of primary human renal mesangial cells with HTNV. HRMCs were inoculated with HTNV (MOI 1) and analyzed for N protein expression by immunofluorescence (scale bar: 100 μm) (**A**) and Western blot (**B**). Amount of infected cells was determined by counting N protein expressing cells (**C**). Infectious units (IU) released from HRMCs were quantified by single round infection assay on Vero E6 cells (**D**). Mean ± SD of three independent experiments is shown

To confirm the replication kinetic of HTNV in mesangial cells, we examined HTNV infection of primary human mesangial cells (Fig. 6). In contrast to the cell line, it is not possible to cultivate primary HRMCs for eight days, therefore the replication was monitored for six days post infection. As observed for CIHGM-1 cells, initial infection of HRMCs with HTNV was very efficient. HTNV N protein was detected in lysates and the number of infected cells comprised 44.16% ± 8.45% at 2 dpi and was subsequently decreasing to 12.21% ± 11.22% at day six post infection (Fig. 6A, B and C). Infectious particles in supernatants were detected by reinfection assay at day two, four and six (Fig. 6D). Together, the results for infection rates with different orthohantavirus strains in the mesangial cell line are in accordance with those from primary cells.

## Discussion

Infections with orthohantaviruses are characterized by a virus-specific clinical picture. Differences exist in the specific organ manifestation between American HCPS- and Eurasian HFRS-causing species, but also in disease severity within the pathogenic species. Viral factors that are responsible for the broad range of clinical picture are not well understood. For infections with Eurasian orthohantaviruses no adequate small animal model exists that recapitulates human disease [53] and research on the underlying pathological mechanisms of HFRS mainly depends on the use of in vitro cell culture models. Kidney cells represent target cells and infection may directly contribute to orthohantavirus-induced acute kidney injury (AKI). The replication kinetics of PUUV and HTNV as pathogenic members of orthohantaviruses differ in mesangial cells. In contrast to HTNV infection, we observed an increase of PUUV-infected mesangial cells over time. The high percentage of mesangial cells initially infected by HTNV may contribute to the pathogenicity of HTNV by viral spread to other renal cells, release of soluble factors and immune-mediated injury. Activation of the immune system early after infection may impact the clinical course of HTNV compared to PUUV and TULV, which are characterized by lower infection rates. The initially robust infection followed by a decrease of HTNV-infected cells may be the result of virus-specific early suppression and subsequent strong induction of the innate immune system. As shown for several orthohantaviruses and cell types, infection causes characteristic changes of transcription profiles resulting in differentially regulated immune responses and specific courses of replication [38, 54–56]. These alterations of the host cell transcriptome and induction or suppression of antiviral response may be linked to the pathogenicity of orthohantaviruses. Mesangial cells possess immune-like characteristics, such as phagocytosis, presentation of antigens, release of cytokines and chemokines [57, 58]. They play a key role in the response to glomerular injury and may therefore control virus infections in a way that was not observed in other renal cell types. It seems that effects are highly specific for cell types and viruses. This emphasizes the importance of using relevant cell culture models to analyze orthohantavirus-induced AKI. The substantial differences in the replication kinetics of high-, moderate- and low-pathogenic orthohantavirus species are of special interest. The role of the viral receptor has also to be elucidated in more detail. TULV as non-pathogenic virus may enter cells via integrin β1, whereas pathogenic orthohantaviruses are described to use β3 integrins for entry [49, 50]. However, the list of receptors described to mediate entry of orthohantaviruses is continuously growing. Several proteins such as CD55, gC1qR, protocadherin-1, TIM-1 (T-cell immunoglobulin and mucin domain 1), have been identified to play a possible role in the entry of various orthohantavirus species [52, 59–62]. Entry studies use various methods, cell types and viruses to identify receptors. It is possible, that orthohantaviruses enter target cells by different ways and differences between virus species and cell types may exist.

Remarkably, we observed the release of infectious units from primary cells and the cell line for all three viruses. For TULV- and HTNV-infected CIHGM-1 cells the release is limited to day 2 and 4 post infection. In addition, the viral spread and replication kinetics differ enormously between PUUV, TULV and HTNV although they release comparable titers at day two post infection. As observed in previous studies, there is no correlation between percentage of infected cells and the titers of infectious particles in the supernatant [38]. Viral titers were determined on interferon-deficient Vero E6 cells and possibly TULV and HTNV particles are not infectious or replication-competent in mesangial cells due to activation of antiviral mechanisms in human cells. Variations in the induction of the innate immune response may result in the release of defective particles and may be responsible for effective or poor viral spread of orthohantavirus species as described for other cell types [38, 55, 63]. The identification of the underlying mechanisms of these cell- and virus-specific replication characteristics will help to understand the pathogenesis of orthohantavirus disease.

Orthohantaviral studies in human kidney cells are still sparse and use mostly primary cells. Primary cells have several disadvantages: since the cell number is limited, only few experiments are possible, and purchase and media are expensive. In addition, donor-specific effects may influence the results, because orthohantaviral infections show a broad variation in the clinical outcome due to patient-specific characteristics [64]. Therefore, future research may be facilitated by adequate kidney cell lines. Results concerning receptor expression, infection rates, viability, and adhesion in the mesangial cell line CIHGM-1 correspond to the situation observed in infected primary mesangial cells. However, infected CIHGM-1 cells demonstrate a reduced migration capacity that was not observed in primary mesangial cells. Glomerular filtration depends on the structural integrity, and maintenance of the filtration barrier requires a regulated motility and adhesion of glomerular cells [65]. In animal models exhibiting altered migration and adhesion capacity of mesangial cells, glomerular development and repair is disturbed [66, 67]. Infected mesangial cells with decreased motility may contribute to kidney injury. The effect of infection on mesangial motility has to be examined in more detail, because it was observed in the cell line but not in primary cells. This difference may be due to the immortalization process of the cell line. Possible morphological and functional changes are a consequence of dedifferentiation and a disadvantage of cell lines. Therefore, cell lines cannot completely replace primary cells.

Together, CIHGM-1 cells represent a suitable in vitro cell culture model concerning mesangial permissiveness and orthohantaviral replication. Using renal cell lines may open the possibility to analyze the orthohantaviral receptor usage, replication cycle, and its differences between species involved in the pathogenesis of AKI.

## Conclusion

The replication kinetics of HFRS-causing orthohantaviruses with low, moderate and high pathogenicity demonstrate strong differences in human glomerular mesangial cells. The ability of orthohantaviruses to replicate in human kidney cells plays a central role in the pathogenesis of acute kidney injury in HFRS. The infection of mesangial cells may influence the viral spread to other target cells within the kidney and may also influence the local immune response by release of soluble factors. Identification of virus- and cell type-specific determinants, which are responsible for the observed differences, will provide useful insights in the underlying mechanisms of orthohantavirus-induced AKI. The use of a mesangial cell line will facilitate this future work.

## Data Availability

Data used to support the findings of the work are included in the article. Raw data will be made available by the corresponding author on reasonable request.

## Abbreviations

AKI: acute kidney injury
CIHGM: conditionally immortalized human glomerular mesangial cells
CIHP: conditionally immortalized human podocytes
dpi: days post infection
HFRS: hemorrhagic fever with renal syndrome
HRMCs: human renal mesangial cells
HTNV: Hantaan virus
PUUV: Puumala virus
TULV: Tula virus
